# Usual dietary treatment of gestational diabetes mellitus assessed after control diet in randomized controlled trials: subanalysis of a systematic review and meta-analysis

**DOI:** 10.1007/s00592-018-1238-4

**Published:** 2018-10-17

**Authors:** Apolonia García-Patterson, Montserrat Balsells, Jennifer M. Yamamoto, Joanne E. Kellett, Ivan Solà, Ignasi Gich, Eline M. van der Beek, Eran Hadar, Eurídice Castañeda-Gutiérrez, Seppo Heinonen, Moshe Hod, Kirsi Laitinen, Sjurdur F. Olsen, Lucilla Poston, Ricardo Rueda, Petra Rust, Lilou van Lieshout, Bettina Schelkle, Helen R. Murphy, Rosa Corcoy

**Affiliations:** 1 Institute of Biomedical Research (IIB Sant Pau), Hospital de la Santa Creu i Sant Pau, Barcelona, Spain; 2 Department of Endocrinology and Nutrition, Hospital Mútua de Terrassa, Terrassa, Spain; 3 Division of Endocrinology, Department of Medicine, University of Calgary, Calgary, Canada; 4 Norfolk and Norwich University Hospitals, Norfolk, UK; 5 Iberoamerican Cochrane Centre, Hospital de la Santa Creu i Sant Pau, Barcelona, Spain; 6 CIBER Epidemiología y Salud Pública (CIBERESP), Instituto de Salud Carlos III, Madrid, Spain; 7 Department of Epidemiology, Hospital de la Santa Creu i Sant Pau, Barcelona, Spain; 8 Department of Pharmacology and Therapeutics, Universitat Autònoma de Barcelona, Bellaterra, Barcelona, Spain; 9 CIBER Salud Mental (CIBERSAM), Instituto de Salud Carlos III, Madrid, Spain; 10 Nutricia Research, Utrecht, The Netherlands; 11 Department of Pediatrics, University Medical Centre Groningen, University of Groningen, Groningen, The Netherlands; 12 Rabin Medical Center, Tel-Aviv University, Tel-Aviv, Israel; 13 Nestlé Research Center, Lausanne, Switzerland; 14 Obstetrics and Gynecology, University of Helsinki, Helsinki, Finland; 15 Helsinki University Hospital, Helsinki, Finland; 16 Institute of Biomedicine, University of Turku, Turku, Finland; 17 Turku University Hospital, Turku, Finland; 18 Statens Serum Institut, Copenhagen, Denmark; 19 King’s College, London, UK; 20 R&D Department, Abbott Nutrition, Granada, Spain; 21 Department of Nutritional Sciences, University of Vienna, Vienna, Austria; 22 ILSI Europe a.i.s.b.l., Brussels, Belgium; 23 Cambridge University Hospitals NHS Foundation Trust, Cambridge, UK; 24 Norwich Medical School, University of East Anglia, Norwich, UK; 25 Department of Medicine, Universitat Autònoma de Barcelona, Bellaterra, Barcelona, Spain; 26 CIBER Bioengineering, Biomaterials and Nanotechnology (CIBER-BBN), Instituto de Salud Carlos III, Madrid, Spain; 27 Servei d’Endocrinologia i Nutrició, Hospital de la Santa Creu i Sant Pau, Sant Antoni M Claret 167, 08025 Barcelona, Spain

## Introduction

The prevalence of GDM is on the rise in relation to an increase in predisposing maternal characteristics. The increase is more marked with application of IADPSG-WHO 2013 criteria [1], with very high rates in special populations [2].

Lifestyle modifications are the first step in the management of GDM and medical nutrition therapy is an essential component of it. Maternal diet should provide adequate energy intake to promote maternal and fetal health, help achieve glycemic goals and be culturally appropriate and individualized [3]. DRI for normal weight pregnant women should be taken into account: provide no increase in energy requirement during the first trimester, + 340 kcal/day in the second trimester and + 452 kcal/day in the third; provide > = 175 g carbohydrate/day, 71 g protein/day and 28 g fiber/day; and have an acceptable energy macronutrient distribution range (45–65% of energy from carbohydrates, 20–35% of energy from fat, 10–35% of energy from protein). However, little is known about the characteristics of diets consumed by women with GDM.

We aimed to characterize the dietary intake of women with GDM in usual clinical care.

## Study protocol

We recently performed a systematic review and meta-analysis on RCTs addressing modified dietary interventions for the treatment of GDM and providing information on maternal glycemic control and birthweight-related variables [4] (published protocol: PROSPERO CRD42016042391).

As a post hoc analysis, we have now examined the composition of diets used by the control group to characterize diets advised for treatment of GDM in usual clinical care. Data on ten dietary characteristics (kcal/day, % of energy provided by carbohydrates, protein, fat, monounsaturated fat, saturated fat and polyunsaturated fat, grams of fiber/day, glycemic index and load) were collected. Glycemic index is defined as the incremental area under the blood glucose curve following the ingestion of a test food, expressed as percentage of the corresponding area following an equivalent load of a reference carbohydrate. The glycemic load takes into account the amount of food intake.

We have used STATA 14.0 and a random effects model to pool the diet characteristics. Heterogeneity was assessed using *I*^2^ statistics and Cochran’s *Q* test. A figure displaying worldwide carbohydrate energy contribution was constructed using the carbohydrate intake of studies providing this information (filled circle) and carbohydrate advice (open circle) when intake was not available.

## Results

Out of 3660 records identified through database search and 128 from other sources, 126 full-text articles were assessed for eligibility and 18 studies were included in the meta-analysis of glycemic control and birthweight-related variables [4]. Thirteen of these studies provided quantitative information on one or more diet characteristics and were included in the current meta-analysis and graphical display. The carbohydrate intake was the diet characteristic most frequently reported (*N* = 12). Other studies only reported diet recommendations and the four of them giving data on carbohydrate advice were included for graphical display.

In the 13 studies included in the current analysis, the modified dietary intervention used for treatment of GDM was as follows: a low glycemic index diet (*N* = 4), a low carbohydrate diet (*N* = 1), Dietary Approaches to Stop Hypertension (*N* = 3), modification of dietary fat (*N* = 2), soy protein enrichment (*N* = 1), behavioral intervention (*N* = 1), and calorie restriction (*N* = 1). The information in the intervention arm is not used in the current analysis.

Pooled estimates on control diet characteristics are summarized in Table 1. High heterogeneity was observed in the ten diet characteristics (*I*^2^ ranging from 94.8 for glycemic load to 99.2 for % of energy from polyunsaturated fat; *p* for heterogeneity < 0.001 for all of them).

**Table 1:** Characteristics of control diet in RCTs addressing modified dietary interventions for GDM treatment (pooled estimates)

Characteristic	*N* studies	Median	CI 95%	*I* ^2^	*P* heterogeneity
Energy (Kcal/day)	10	2094.0	1931.9–2256	98.1	< 0.001
% of energy from carbohydrates	12	49.1	45.1–53.1	98.5	< 0.001
% of energy from proteins	11	19.0	17.1–20.9	98.5	< 0.001
% of energy from total fat	11	31.5	28.6–34.4	97.7	< 0.001
% of energy from saturated fat	7	9.6	8.3–10.8	96.6	< 0.001
% of energy from polyunsaturated fat	6	9.5	8.3–10.7	99.2	< 0.001
% of energy from monounsaturated fat	3	10.1	6.1–14.1	96.8	< 0.001
Glycemic index	4	54.3	51.2–57.5	98.1	< 0.001
Glycemic load	3	122.3	108.1–136.4	94.8	< 0.001
Fiber (g/day)	10	21.6	18.9–24.2	98.0	< 0.001

**Fig. 1:**
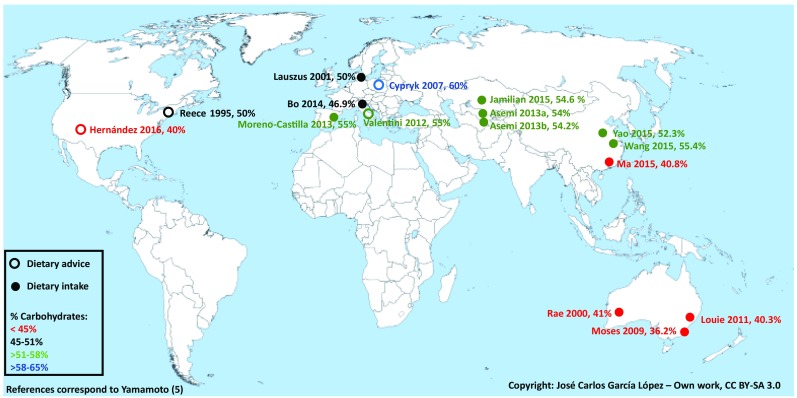
Energy derived from carbohydrates in control diets of RCTs addressing modified dietary interventions for treatment of GDM by trial site

The dietary carbohydrate content of control diets in individual trials is displayed in Fig. 1. Carbohydrate contribution to energy intake ranged from moderate restriction (36.2% in Australia) to the upper range of the acceptable macronutrient distribution range (60.0%, Poland).

## Discussion

In this subanalysis addressing control diets in RCTs on modified dietary interventions for GDM, we observed a high heterogeneity in the ten analyzed characteristics. This information has not been previously reported.

The figures of carbohydrate content of control diets parallel with some exceptions the diet composition in the background population according to FAO statistics [5] with the incorporation of some degree of carbohydrate restriction.

It is of note that specific dietary recommendations with regard to energy-yielding nutrients are lacking for treatment of GDM. A limitation of the current analysis is that we did not perform a specific systematic review and meta-analysis to address this topic but a subanalysis of a previous one [4]. However, current results can serve as an estimation of diets usually advised to women with GDM. Another limitation is that the number of meals and snacks was not addressed.

We conclude that control diets used in RCTs addressing modified dietary intervention in women with GDM display marked heterogeneity in all analyzed characteristics, probably reflecting the diet properties of the background population. This is desirable from the cultural and socioeconomic point of view, but may have an impact on the response to nutritional management of GDM and should be addressed in future research.

## Abbreviations

DRI: Dietary reference intakes
GDM: Gestational diabetes mellitus
RCT: Randomized controlled trial

## References

[1] Meek CL, Lewis HB, Patient C, Murphy HR, Simmons D (2015). Diagnosis of gestational diabetes mellitus: falling through the net. Diabetologia.

[2] Egan AM, Vellinga A, Harreiter J (2017). Epidemiology of gestational diabetes mellitus according to IADPSG/WHO 2013 criteria among obese pregnant women in Europe. Diabetologia.

[3] Metzger BE, Buchanan TA, Coustan DR,et al (2007) Summary and Recommendations of the Fifth International Workshop-Conference on Gestational Diabetes Mellitus. Diabetes Care 30:S251–260. 10.2337/dc07-s225 10.2337/dc07-s225 17596481

[4] Yamamoto JM, Kellett JE, Balsells M (2018). Gestational diabetes mellitus and diet: a systematic review and meta-analysis of randomized controlled trials examining the impact of modified dietary interventions on maternal glucose control and neonatal birth weight. Diabetes Care.

[5] FAO. ChartsBin statistics collector team 2011, Contribution of Carbohydrates in Total Dietary Consumption, ChartsBin.com, viewed 6th May, 2018, http://chartsbin.com/view/1154

